# Non-cadaveric spine surgery simulator training in neurosurgical residency

**DOI:** 10.1016/j.xnsj.2024.100573

**Published:** 2024-11-09

**Authors:** Paul Pöser, Robert Schenk, Hannah Miller, Ahmad Alghamdi, Adrien Lavalley, Katharina Tielking, Nitzan Nissimov, Anton Früh, Denny Chakkalakal, Victor Patsouris, Tarik Alp Sargut, Robert Mertens, Ran Xu, Peter Truckenmüller, Kiarash Ferdowssian, Judith Rösler, David Wasilewski, Claudius Jelgersma, Anna Roethe, Aminaa Sanchin, Peter Vajkoczy, Thomas Picht, Julia Sophie Onken

**Affiliations:** a Department of Neurosurgery, Charité - Universitätsmedizin Berlin, corporate member of Freie Universität Berlin, Humboldt-Universität zu Berlin, and Berlin Institute of Health, Berlin, Germany; b Berliner Simulations- und Traingszentrum (BeST), Berlin, Germany; c Cluster of Excellence: “Matters of Activity. Image Space Material,” Humboldt University, Berlin, Germany; d Berlin Institute of Health, BIH Academy, (Junior) Clincian Scientist Prgram, Charité-Universitätsmedizin Berlin, Berlin, Germany

**Keywords:** Exoscope, Microsurgery, Neurosurgery, Non-cadaveric spine surgery training, Residency

## Abstract

**Background:**

Spine surgical training faces increasing challenges due to restricted working hours and greater sub specialization. Modern simulators offer a promising approach to teaching both simple and complex spinal procedures. This study evaluated the acceptance and efficacy of spine simulator training using a lumbar herniated disc model tested by 16 neurosurgical residents (PGY-1-6), and compared 3D and 2D teaching methods.

**Methods:**

Sixteen residents utilized the Realists RealSpine L4/L5 disc simulator with both microscope and exoscope. A mixed-methods analysis assessed the efficacy and acceptance of the training. Six PGY-1 residents participated in a learning curve study, divided into exoscopic and microscopic cohorts. Each group watched a tutorial in either 3D or 2D, followed by 3 surgical sessions. Endpoints included surgical progress within 30 minutes and complication rates. Microsurgical skills and mental concepts were evaluated on a numeric Likert Scale.

**Results:**

Participants rated the simulator training favorably, with a median score of 8/10 across 6 categories. The learning curve study showed a 30% improvement in microsurgical performance. The completion rate of herniated disc removal increased from 50% at T2 to 100% at T3 and T4. Significant improvement in mental concept was observed (p=.035), with slightly better consolidation in the exoscope group. Self-assessments revealed significantly improved skills across all participants.

**Conclusions:**

Spine simulator training was well-received and resulted in improvements in both mental concept and microsurgical performance, with enhanced outcomes in the 3D teaching/exoscope group. This study supports the integration of spine simulators into spine surgical residency, particularly for early-stage training, to improve both cognitive and practical surgical skills.

## Graphical abstract

**Figure:**
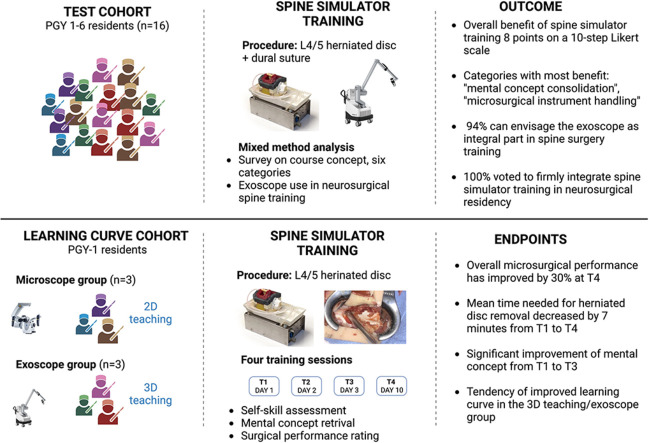

## Introduction

Current trends in clinical structures, such as restrictive working hours, personnel restraints, and tight operational schedules, are limiting the training opportunities available to neurosurgical residents. Hence, sustainable concepts in education are being sought to compensate for limited operating room (OR) exposure in a highly demanding field like neurosurgery. Simulator training for surgeons offers a way to provide a more efficient, feasible, and time-effective way to acquire skills compared to well-established cadaver courses.[1], [2], [3], [4], [5], [6] The use of simulators allows training outside of clinical schedules and offers the possibility of acquiring surgical skills in a safe environment.^4^^,^[7], [8], [9]. Previous studies evaluated the effectiveness of a simulator-based curriculum for training first-year neurosurgical residents in craniotomy procedures. They found that simulator training led to significant improvements in technical skills and reduced operative time in the OR^2^^,^[10], [11], [12], [13], [14], [15], [16], [17].

Recent technological advancements allow now for highly realistic imitation of spinal pathologies with imitation of muscular and epidural bleeding and cerebrospinal fluid (CSF) leakage.^18^ Until today, there is no evidence on the overall acceptance of non-cadaveric spine simulator training in neurosurgical residency, its effectiveness in achieving microsurgical competency, and its impact on the learning curve of post-graduate neurosurgical residents.^19^

With this study, we evaluate the acceptance and efficacy of a non-cadaveric, simulation-based spine surgery training among neurosurgical residents (n=16 PGY-1-6). To further prove the impact of spine simulator training on the learning curve of PGY-1 residents, mental concept consolidation, objective and subjective surgical skills/performances were evaluated at 4 consecutive training sessions. Additionally, we used the training to investigate the acceptance of the exoscope in spine surgery and the effect of 3D-based teaching/exoscope use compared to 2D-based teaching/microscope use. The results of this study provide valuable insights into the effectiveness of non-cadaveric spine simulator training in neurosurgery and will inform the development of future curricula.

## Methods

### Noncadaveric spine surgery simulator

We chose the *Realists RealSpine* (Realists Training Technologies, Leipzig, Germany) herniated disc model L4/5 for an interlaminar approach in this study.^20^ This model has been validated in previous studies and has already been widely adopted in the spine surgery community.[20], [21], [22], [23] It features a realistic representation of all key anatomical structures, including the epidural fat, haptically realistic bone, ligamentum flavum, and herniated disc.^24^ One of the key features of the *Realists RealSpine* herniated disc model is the ability to simulate bleeding from the lamina of the vertebral arch, muscle, and epidural space at varying intensities. Additionally, it can simulate a CSF leak in case of dural injury.

### Test cohort and study setup

The surgical simulators were made available to 16 neurosurgical residents in their PGY- 1-6 (**test cohort**) and evaluated by mixed method analysis. The experience level of the test cohort with herniated disc surgery and dural tear repair is given in Table 1. Each participant explored the model with the exoscope and microscope. Participants purposely induced a dural injury and sewed it up. After the training, the test cohort completed a survey evaluating their experience and subjective perceptions of the noncadaveric spine model in 6 categories (“Self-confidence in the OR,” “Consolidation of the mental concept,” “Instrument knowledge,” “Anatomic orientation,” “Handling of microsurgical instruments,” “Microscope/exoscope handling”) on a 10-step Likert scale ranging from 1= no benefit to 10= strong benefit. The participants were asked if they could picture the exoscope as an integral part of neurosurgical residency (yes/no answer). Furthermore, the test cohort was asked about the feasibility of integrating a noncadaveric spine surgery course into the neurosurgical curriculum (yes/no answer). Specific questions on the course setting with emphasis on required personnel resources and digital teaching material were asked using a 10-step Likert scale ranging from 1= no benefit to 10= strong benefit. Lastly, we asked for the optimal timing of such a course during residency (PGY 1-6).

**Table 1:** Experience level of the learning curve cohort and test cohort with lumbar herniated disc surgery and dural tear repair.

Experience level	**Learning curve cohort (n=6)**		**Test cohort (n=16)**	
	HD surgery		HD surgery	Dural tear repair
	Exo. (n=3)	Mic. (n=3)		
**Theory only**	-	1	-	2
**Assistance only**	1	-	4	9
**Little practical experience** Soft tissue preparation	2	2	5	3
**Some practical experience** Preparation of the flavum lig.	-	-	3	-
**Completion of the procedure under supervision**	-	-	3	1
**Completion of the procedure without supervision**	-	-	1	1
**Previous experience with exoscope**	0	0	5	

Exo, exoscope; HD, herniated disc; lig, ligament; mic, microscope.

### Learning curve cohort and study set-up

The **learning curve cohort** consisted of 6 PGY-1 neurosurgical residents without prior surgical experience in performing a lumbar herniated disc surgery. *A priori*, all participants completed a baseline surgical skill assessment (Table 1).

The participants were then assigned to matching groups to undergo training with the exoscope or the microscope. On day 1 (T1), a video tutorial covering the key steps of our department's standard for lumbar herniated disc surgery was presented to the participants. The participants of the exoscope group were trained with a 3D video, the microscope group was trained with a 2D video. The participants had the opportunity to ask questions and discuss the procedure with an attending neurosurgeon on site.

The practical part of the study consisted of 3 days during which operations were performed on the simulator. On day 1 (T2), 2 (T3), and 10 (T4) after the video tutorial, the participants performed the surgical procedure for 30 minutes under supervision with either the exoscope or the microscope. During surgery, the participant had the attending as their surgical assistant. The attending only intervened when prompted by the resident or the procedural progress stalled. Number of inquiries by the trainee and interventions by the tutor/attending neurosurgeon throughout the procedure were recorded.

Such interventions were classified as minor (eg incorrect instrument selection and instrument handling) or major interventions/complications (eg anatomic malorientation, residual herniated disc material after 30 minutes surgery time, dural tear).

To assess the learning curve throughout the spine surgery training course, each participant was asked to write down the **mental concept** of the procedure prior T1, T2, T3 and T4. The written mental concept of the procedure was compared to the institutional gold standard, which involved 41 steps from skin incision to closure that have been mentioned in the teaching video. The written mental concepts were anonymized and reviewed by 2 blinded reviewers. Each correct step was awarded 1 point. Results were analyzed longitudinally per individual and compared between the exoscope and microscope groups.

Each surgical procedure was recorded for later video analysis for **objective microsurgical performance rating** by the attending neurosurgeon and 1 additional member of the study group. A total of 18 categories were evaluated on a 10-step Likert scale (Table 2).

**Table 2:** Objective microsurgical performance rating based on 18 categories, rated on a 10-step Likert scale.

Mental aspects	Surgical performance	Manuel skills
The resident…		
…follows an agreed, logical sequence or protocol for the procedure	…considerately incises flavum ligament	…appropriately handles microscope/exoscope
…proceeds at an appropriate pace with economy of movement	…fenestrates level L4/5 safely	…consistently handles tissue well with minimal damage
…anticipates and response appropriately to variation e.g. anatomy	…identifies the relevant nerve root and local pathology	…uses instruments appropriately and safely
…deals calmly and effectively with untoward events/complications	…clearly preparates nerve root	…controls bleeding promptly by an appropriate method
…uses assistance to the best advantage at all times	…inspects intervertebral disc space properly	
… has a comprehensive knowledge of the instruments	…safely retracts nerve root and evacuates relevant herniated disc material	
	…appropriately flushes situs	
	…ensures that there is no remaining nerve root compression prior closure	

Prior T1 and after T4, participants conducted a **self-skill assessment** comprising 13 questions about their general and spine-specific surgical skills, rated on a 5-step Likert scale (1= insufficient to 5= very good).

All evaluations including microsurgical skills, mental concepts, and self-skill assessments, were conducted anonymously by attending neurosurgeons. Videos were reviewed, and mental concepts were evaluated based on a gold standard checklist. No third-party software was used for evaluation.

### Data management, statistical analysis, and graphic design

Study data were collected and managed using REDCap electronic data capture tools hosted at Charité – Universitätsmedizin Berlin. Statistical analysis of this exploratory study was performed using GraphPad Prism 9 (GraphPad Software, San Diego, CA, USA) and SAS Version 9.4 (SAS Institute, NC, USA). For the descriptive analysis of differences between 2 groups in nominal variables, unpaired T-test was applied. For the descriptive analysis of more than 2 groups, 1-way ANOVA combined with Bonferroni's multiple comparison test or Kruskal Wallis test with Dunn's test for multiple comparisons was used, depending on the data`s normal distribution. Results of the mental concept and scores from objective microsurgical performance rating are given in sum scores. Mean values and standard deviation (SD) of the respective cohort were compared at different time points using Chi-square tests. For graphic design, BioRender (Science Suite, Toronto, ON, Canada) was used.

### Ethical statement

This study was conducted according to the ethical principles of medical research involving human subjects according to the Declaration of Helsinki and its later amendments. All participants gave their written consent to participate in the study. Comparison study of neurosurgical standard procedures between ocular-based surgical microscope and exoscopic OrbEye Application number: EA4/068/21.

### Theory

• Positioned at the intersection of anatomical exploration and the development of surgical motion.
• memory through simulation technology, our study investigates the role of simulators in spine.
• surgery education. Our study dives deeper into the relationship between the theoretical.
• knowledge of spine surgery and the innovative use of simulation technology, with a focus on.
• comparing exoscope and microscope applications. Our Objective is to create a course concept.
• that allows for enhanced learning and practice of surgical procedures. Transitioning towards.
• practical validation, our investigations, anchored by various quantitative methodologies, provide.
• substantial empirical support for the effectiveness of the simulator training. This provides insights into the educational potential of noncadaveric surgical training and lays the foundation for future concept creation.

## Results

### Evaluation of the non-cadaveric spine simulator training by 16 neurosurgical PGY-1-6 residents

**Fig. 1:**
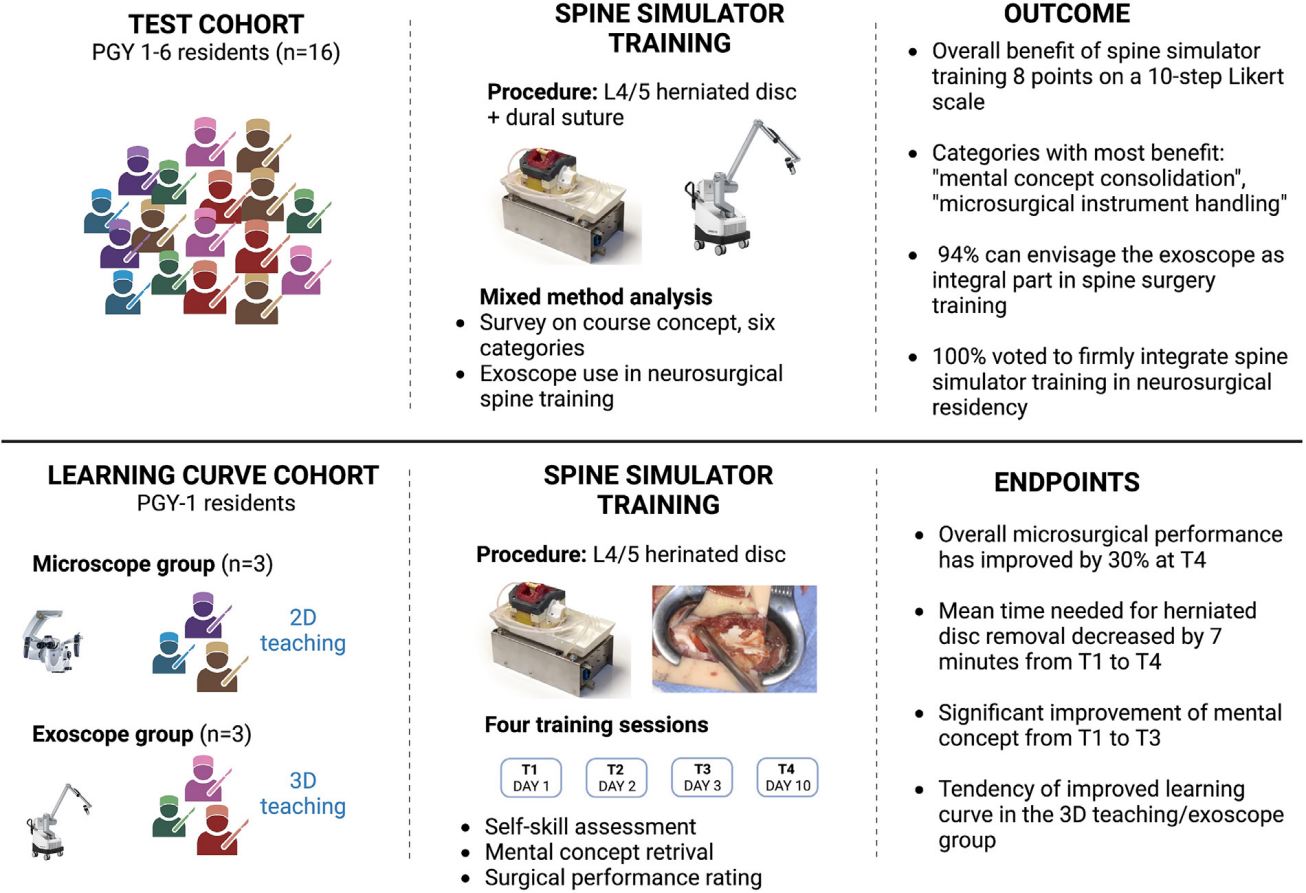
Graphical abstract.

The model was tested by a total of 16 neurosurgical residents in their PGY- 1-6 of neurosurgical training (**test cohort**). Overall, the benefit of the noncadaveric spine surgery training was rated favorable with a median of 8 points on a 10-step Likert scale among all applied categories. The residents identified “consolidation of the mental concept” and “handling of microsurgical instruments” areas in which they would expect the most benefit (Fig. 1A). Concerning the course setup and personnel resources for maintenance of simulator training, the importance of having a tutor on site was strongly supported by most participants (Fig. 1B), whereas the replacement of the tutor with a detailed teaching video was rated controversial (Fig. 1C). The test cohort unanimously reported satisfaction with the training experience and appreciated the possibility of honing their skills and discussing critical steps in the simulation setting. The entire test cohort can envisage simulator training becoming an integral part of the neurosurgical curriculum within PGY-1 and -2.

### Impact of spine simulator training on mental concept formation and on surgical skills in PGY-1 neurosurgical residents

The learning curve study was conducted according to the protocol given in the method section. In brief, each PGY-1 neurosurgical resident watched a video tutorial (T1) in either 2D (microscope group) or 3D (exoscope group), followed by performing the procedure in 3 consecutive sessions, T2, T3, and T4.

### Mental concept consolidation

**Fig. 2:**
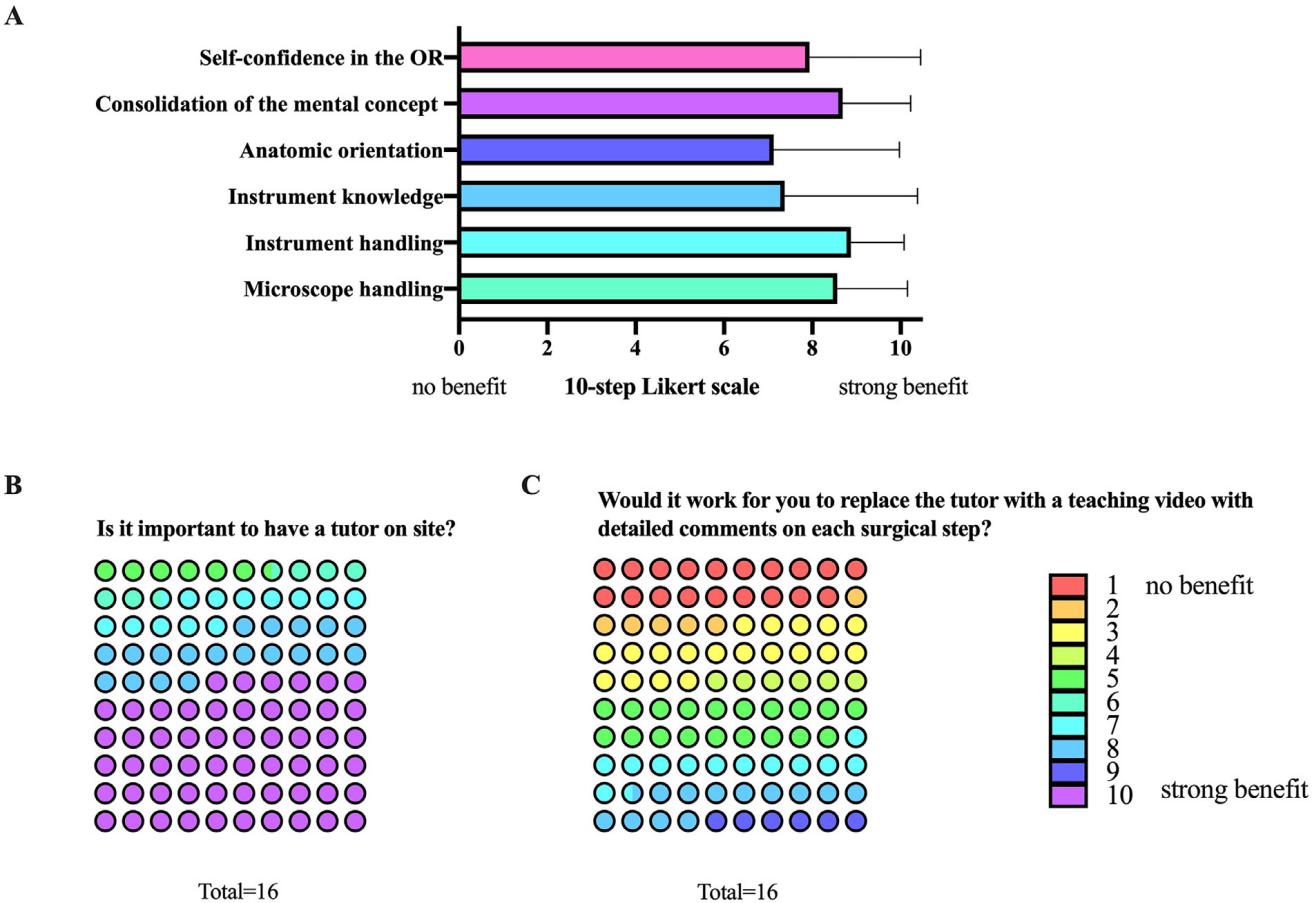
A: Surgical benefits of the cadaver-free spine surgery training course evaluated by the test cohort (n=16) on a 10-step Likert scale, presented as percentages. B+C: Visually presented perceptual results of the feedback received from the 16 individuals. Lig, ligament.

The mental concept was obtained before each training session (T1, T2, T3, and T4) and evaluated. At T1, a mean score of 21 points (SD 8) out of 41 possible points was reached, followed by 24 points (SD 5) at T2, 28 points at T3 (SD 6), and 25 points at T4 (SD 6). All participants showed a steady increase of their score in mental concept consolidation from training session T1 to T3, which has been scheduled on consecutive days. After a training pause of 7 days between T3 and T4, we observed a decreased ability to reproduce the mental concept in 4 out of 6 participants (Fig. 2A). When comparing the mean mental concept scores of the exoscope group to the microscope group, the exoscope group reached a higher mean score at T4 (29 vs. 20 points). However, the student's t-test did not reveal a significant difference between the 2 groups (Fig. 2B).

### Objective evaluation of the microsurgical performance

For objective microsurgical performance rating, we evaluated the surgical progress after the given 30-minute microscope/exoscope time. At T2, 5 out of 6 participants were able to access and remove the herniated disc within 30 minutes. This increased to 100% at T3 and T4. The time from start to subjective completion of removal of the herniated disc is displayed in Fig. 2C for each participant. We observed an overall improvement in the operative time by average of 7 minutes, without statistical significance between the exoscope and microscope group (Fig. 2D). Surgical performance was then objectified based on 18 criteria, which can be assigned to the following categories: “mental skills,” “surgical performance” and “manual skills” (see Table 2). The results show a significant improvement from T2 (mean 109 points) to T3 (mean 145 points) and T4 (mean 158 points) for each participant (Fig. 2E). At T4, the objective microsurgical performance increased by 30% (mean 158 points/200 points). Next, we compared the performance of the exoscope and microscope group. Again, there was a slight superiority of the exoscope group without statistical significance (Fig. 2F). Overall, the improvements were equally evident in all subcategories “mental skills,” “surgical performance” and “manual skills.”

**Fig. 3:**
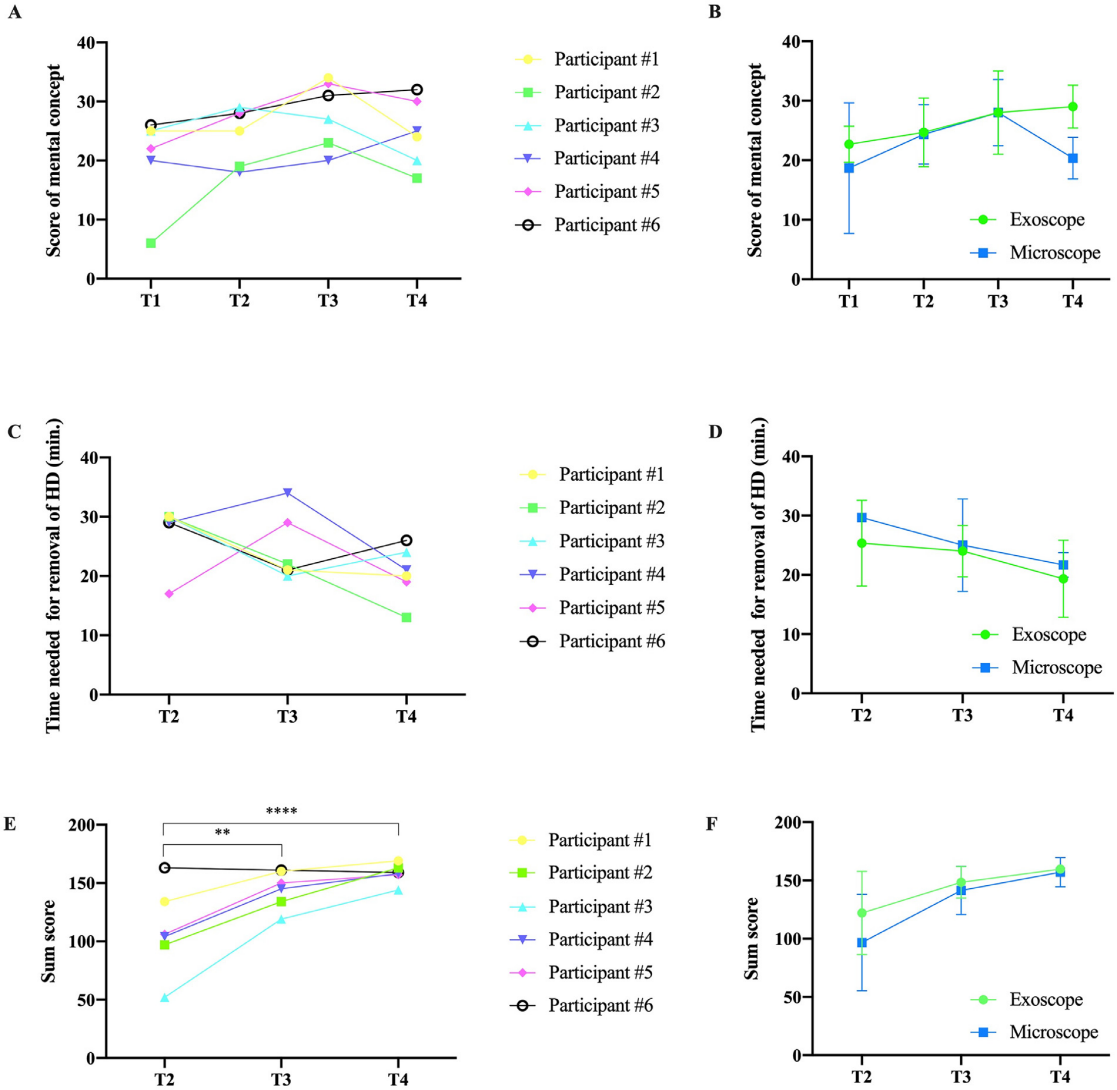
A: Scorings in the mental concept over the course per individual participant. B: Scorings in the mental concept of the exoscope and microscope group. C: Time needed to access and removal of the herniated disc material per individual participant. D: Time needed to access and removal of the herniated disc material in the exoscope and microscope group. E: Objective microsurgical performance rating of each participant displayed in a sum score of 18 categories rated on a 10-step Likert scale (Chi-square test, median sum score T2 vs. T3: p**= .0016, T2 vs. T4:p***<.0001). F: Median sum score of the exoscope and microscope group in objective microsurgical performance rating.

Lastly, we evaluated the frequency of inquiries to the tutor (attending neurosurgeon)/ interventions by the tutor throughout the procedure. Minor interventions (incorrect instrument selection/handling of microsurgical instruments) were recorded at a median 6 times during T2, 3 times during T3, and 3 times during T4. Major interventions were observed 3 times at T2, zero times at T3, and 3 times at T4 (Table 3). Residual herniated disc material was found by the tutor in 50% of the cases at T2, in 0% at T3, and 17% at T4 (Fig. 3A). Causes for minor and major interventions are listed in Table 3, separated in exoscope and microscope groups. Overall, minor interventions were less frequent in the exoscope group compared to the microscope group (9 versus 17).

**Table 3:** Causes for minor and major interventions and complications during spine surgery training course.

	**T2** Mean (range)		**T3** Mean (range)		**T4** Mean (range)	
	Exo.	Mic.	Exo.	Mic.	Exo.	Mic.
**Minor interventions** Incorrect instrument selection/ handling	5 (3-6)	7 (6-9)	2 (1-2)	5 (2-9)	2 (1-3)	5 (2-7)
	**T2** n=		**T3** n=		**T4** n=	
**Major interventions/complications** Anatomic malorientation Residual herniated disc material Dural tear	- 1 -	- 2 -	- - -	- - -	- 1 -	1 1 -

Exo, exoscope; Mic, microscope; n, amount.

### Self-skill assessment of the learning curve cohort

Finally, we analyzed the subjective impact of the non-cadaveric spine surgery training on the surgical skills of each participant. The “surgical skills” self-assessment prior T1 and at T4 revealed significant improvement with an average score of 1.3 pretraining and 3.3 post-training on a 5-step Likert scale (unpaired student´s t-test: p=.0006). Each participant reported an increase in her/his skills in all 5 aspects of the self-assessment, including “opening/removal of the flavum ligament,” “access to the nerve root,” “palpation of the herniated disc,” “removal of the herniated disc” and “inspection of the intervertebral disc space.” Detailed data is displayed in Fig. 3A.

### Benefit of exoscopic training in spine surgery

Spine simulator training was also used to introduce the exoscope to the residents. Although none of the residents had previous experience with the exoscope in spine surgery, 94% of the test cohort could imagine starting or continuing their surgical training with the exoscope after using it with the spine simulator. Although we observed noninferiority of the learning curve results in the exoscope group compared to the microscope group, there is a tendency towards improved mental concept consolidation in the 3D teaching/exoscope group. From the tutor's perspective, the 3D view/exoscope enables improved interaction with the resident.

## Discussion

The principal novel findings of our study demonstrate the efficacy and acceptability of noncadaveric spine simulator training in neurosurgical residency. Our results show that this training modality was well-received by the entire test cohort of 16 PGY 1-6 neurosurgical residents, with all participants envisioning its integration into the neurosurgical curriculum. Most notably, we provide the first evidence of efficacy for non-cadaveric spine simulator training in PGY-1 neurosurgical residents. After only 3 simulator training sessions, we observed significant improvements in 2 key domains: mental concept consolidation and microsurgical performance.

In terms of mental concept improvement, mental concept scores improved steadily from a mean of 21 points at baseline to 28 points by the third session. This improvement reflects an enhanced understanding of the procedural steps and overall surgical strategy.

For microsurgical performance, objective performance, as evaluated by our 18-criteria assessment, increased by 30% from the first to the final session. The duration of the procedure decreased on average by 7 minutes, and by the third session, all participants were able to complete the simulated surgery within the 30-minute timeframe. Additionally, the frequency of tutor interventions decreased over time, indicating improved autonomy and technical proficiency among the participants.

However, the number of training sessions required to feel confident with a particular task may vary depending on the individual. This variability highlights the need for personalized training approaches that can accommodate different learning curves. We propose systematically assessing surgical skills after the completion of 3 consecutive training sessions to ensure a baseline level of competency before residents are allowed to perform their first surgery on a patient. This approach ensures that individualized learning needs are addressed while maintaining patient safety.

Previous studies have reported the benefits of 3D media, augmented reality (AR), and virtual reality (VR) in conveying theoretical knowledge effectively.^14^^,^^30^^,^^31^ Building on this evidence, we integrated 3D-based teaching modalities and exoscope use into our spine simulator training. Our results suggest that the use of 3D teaching and exoscope technology positively impacted training efficacy when compared to 2D teaching and traditional microscope use. One of the key advantages of using the exoscope is that it allows all participants to follow the procedure from the same 3D perspective, which facilitates better anatomical orientation, internalization of procedural steps, and improves team communication during surgery.

Although not statistically significant due to our small sample size, the exoscope group exhibited a tendency towards improved mental concept consolidation and required fewer tutor interventions compared to the microscope group. This suggests that the use of 3D media and the exoscope may further enhance training by allowing for a more comprehensive understanding of surgical anatomy and procedures.

Furthermore, future studies could explore the integration of virtual reality to further enhance the training experience.

Regarding subjective measures, participants reported significant improvements in their surgical skills, as reflected in their self-assessment scores, which increased from an average of 1.3 pretraining to 3.3 post-training on a 5-step Likert scale. This improvement was consistent across all evaluated aspects of the procedure, from opening the flavum ligament to inspecting the intervertebral disc space. The subjective improvement in self-confidence and skill level further supports the effectiveness of noncadaveric spine simulator training.

These findings collectively support the potential of non-cadaveric spine simulator training, particularly when combined with 3D teaching methods and exoscope use, as an effective tool for enhancing both cognitive understanding and practical skills in spine surgery residency programs. The integration of such technologies allows residents to experience surgical procedures more dynamically and collaboratively, which is critical for fostering a deeper understanding of surgical anatomy and technique.

Our findings align with previous research demonstrating the positive impact of simulator-based training on surgical education, particularly in cranial neurosurgery. Prior studies have shown significant improvements in technical skills and reduced operative times following simulator training.^2^^,^[25], [26], [27], [28]This study extends those findings to the context of spine surgery, demonstrating that simulation training can effectively teach both the cognitive and practical aspects of surgical procedures.

The improvements in mental concept consolidation observed in our study align with the model of surgical skill acquisition proposed by Fitts and Posner, which describes cognitive, associative, and autonomous stages of learning (PMID: **17182991**).^29^ Our findings reinforce the idea that structured, repetitive practice offered by simulators accelerates the early stages of learning and skill acquisition, which is crucial in surgical education.

In addition, similar to other studies on minimally invasive spine surgery training, we observed significant improvements after just a few training sessions, suggesting that simulator-based training is an efficient method for developing key surgical skills.^23^ Our study also supports previous findings that 3D teaching modalities, including AR and VR, can enhance theoretical knowledge transfer and improve clarity in anatomical orientation, demonstrating that these technologies have a vital role in surgical education.^1^^,^^12^^,^^14^^,^^30^^,^^31^

Several limitations should be acknowledged. First, the small sample size, particularly in the learning curve cohort, limits the statistical power of the study. Future studies with larger participant pools are needed to validate these findings. Second, we did not provide real-time feedback on mental concept training during the sessions, which might have limited the full potential of improvement. Integrating immediate tutor feedback during training sessions could optimize the learning experience.

Additionally, the short-term nature of the study did not allow us to assess long-term retention of skills or the transferability of these skills to clinical practice. Future longitudinal studies are necessary to determine whether residents retain and apply these skills over time. Finally, the novelty of the simulator might have introduced some bias, as participants may have been motivated by the new technology, which could have influenced their performance.

## Conclusion

This study provides compelling evidence for the integration of noncadaveric spine simulator training into neurosurgical residency programs, particularly in the critical early stages of training. By accelerating mental concept consolidation and enhancing microsurgical performance in a controlled, risk-free environment, simulator-based training addresses key challenges in modern surgical education, including limited operating room exposure and the decreasing availability of cadaveric training. This study is among the first to systematically demonstrate these benefits in spine surgery, underscoring the transformative potential of simulation-based learning.

The findings suggest that repeated, structured practice through simulators not only improves technical proficiency but also enhances overall surgical safety, benefiting both residents and patients. As the complexity of neurosurgical procedures continues to rise, the role of such technology becomes increasingly vital in bridging the gap between theory and hands-on experience.

Institutions are encouraged to develop structured, stage-specific curricula that incorporate simulators for practical skills and mental concept training. Trainees, in turn, must take responsibility for fully engaging with these educational tools and committing time to skill acquisition.

**Fig. 4:**
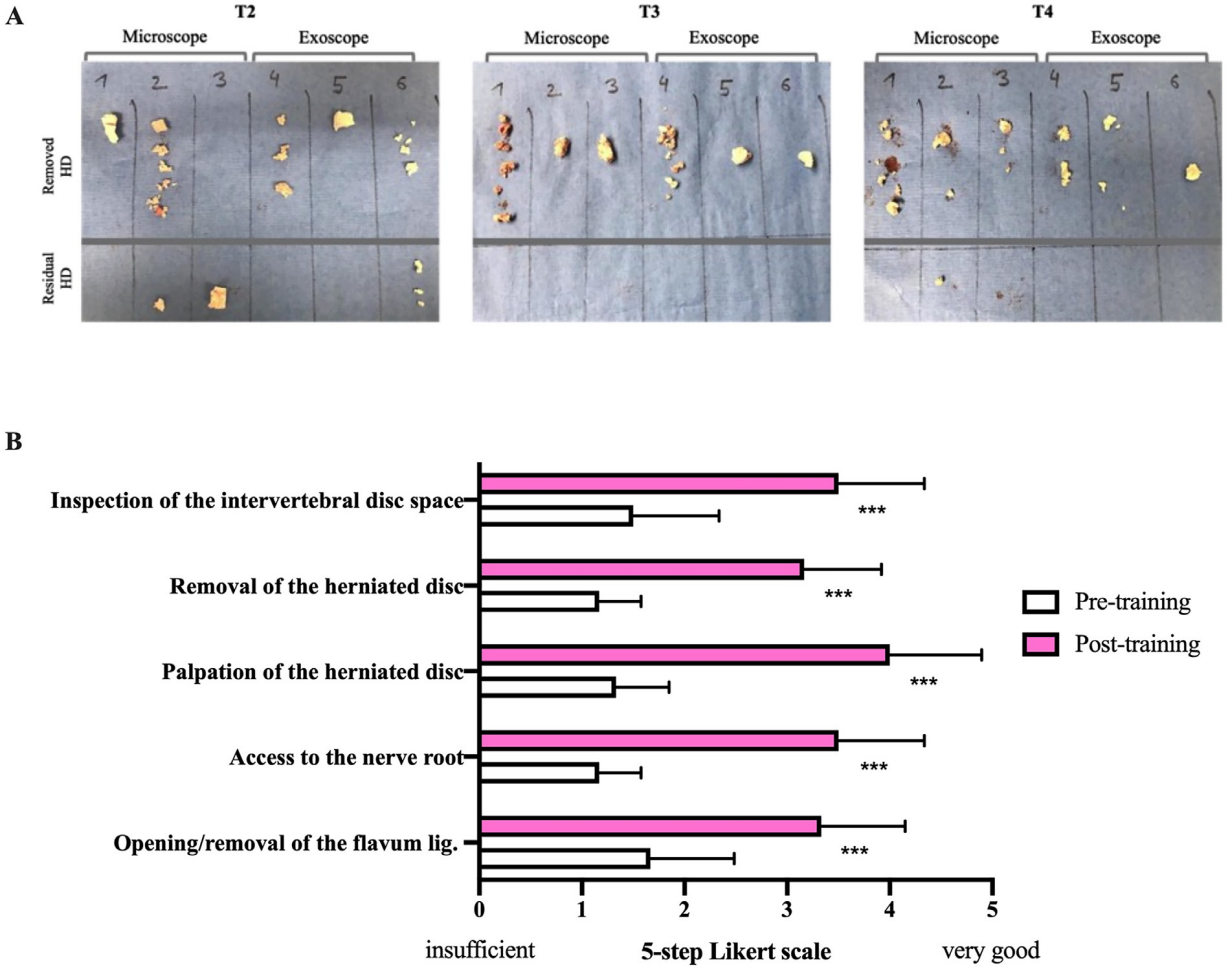
A: Removed and residual herniated disc (HD) material per participant at T2, T3, and T4, separated in microscope and exoscope group. B: Pre-training (T1) and Post-training (T4) self-assessment of the learning curve cohort (n=6) on a 5-step Likert scale. Students t-test, p***<.005.

**Fig. 5:**
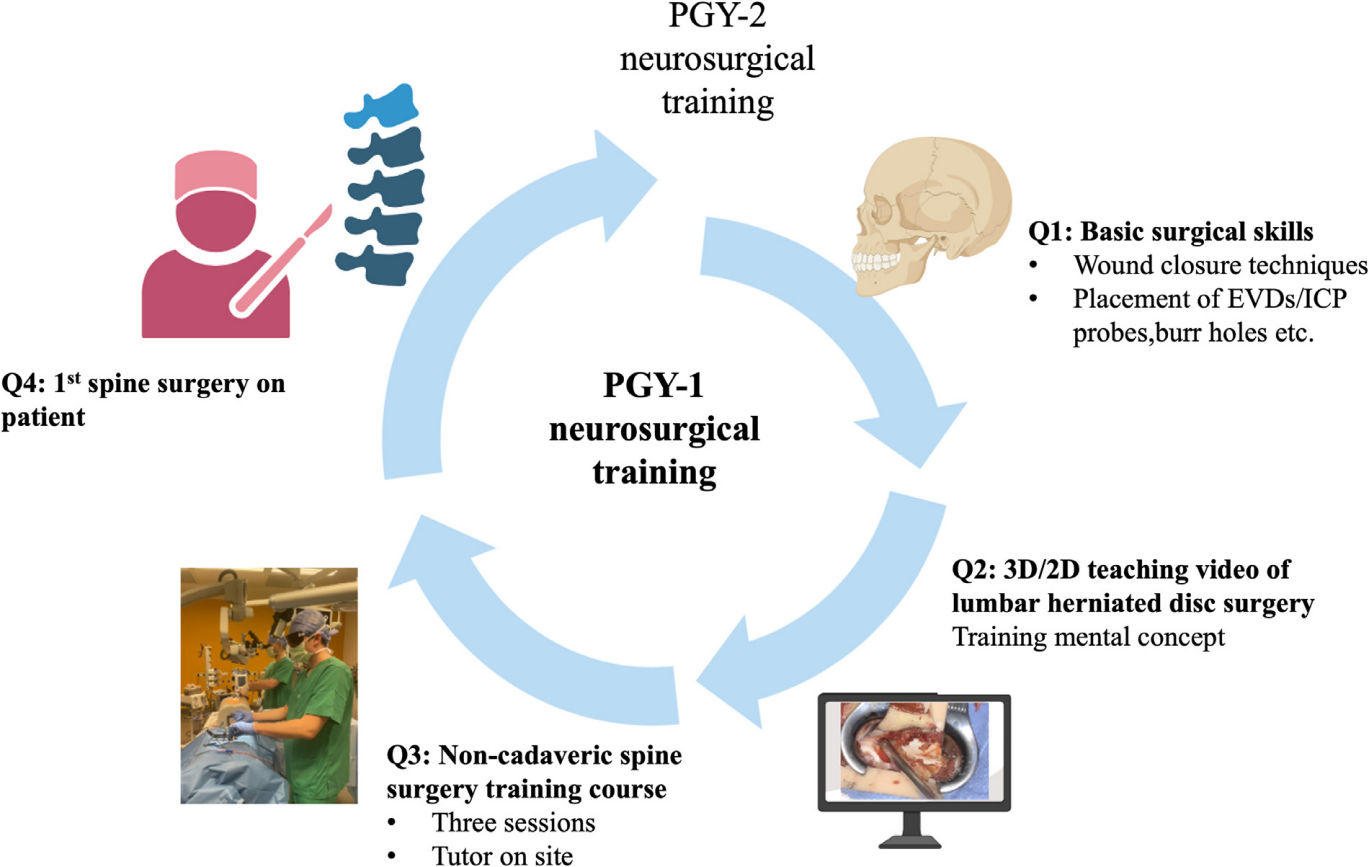
Proposal for a 1st year neurosurgical residency curriculum integrating the non-cadaveric spine course. EVD= external ventricular drainage, ICP, intracranial pressure monitoring; PGY, post graduate year; Q, quarter. Figure created with BioRender.com.

To fully realize the potential of this training method, further research on a larger scale and over extended periods is essential to confirm long-term skill retention and clinical transferability. Integrating emerging technologies, such as virtual reality, could further optimize the training process, making it more immersive and effective. To support widespread adoption, it is critical to secure funding and institutional accreditation for simulation training programs, ensuring that future neurosurgeons are equipped with the skills necessary to meet the demands of modern patient care (Figs. 4 and 5).

## Declaration of competing interest

The authors declare that they have no known competing financial interests or personal relationships that could have appeared to influence the work reported in this paper.
